# Ocular Surface Infection and Antimicrobials

**DOI:** 10.3390/antibiotics11111496

**Published:** 2022-10-28

**Authors:** Debarun Dutta, Fiona Stapleton, Mark Willcox

**Affiliations:** 1 Optometry School Life and Health Sciences, Aston University, Birmingham B4 7ET, UK; 2 School of Optometry and Vision Science, University of New South Wales (UNSW), Sydney 2052, Australia

Infection of the ocular surface can have devastating consequences if not appropriately treated with antimicrobials at an early stage. These infections can lead to blindness through corneal scarring or may lead to the enucleation or evisceration of the globe. Treatment often needs to be fast, empirical and based on disease presentation. However, infection by different microbes (bacteria, fungi, viruses or protozoa) can manifest with similar signs and symptoms, so initial treatment may have to be changed. Furthermore, microbes causing infections are showing increasing resistance to antimicrobials. These delays can worsen outcomes.

This Special Edition was designed to highlight current research in this field, with particular emphasis on the antimicrobial resistance of ocular isolates and developing new ways to prevent or treat ocular infections. Eleven papers have been published in this Special Edition. Their topics range from examining the types of *staphylococci* that cause ocular infections to the development of potentially new ways of treating ocular infections with antimicrobial peptides or predatory bacteria.

Four papers examined the types of *staphylococci* causing ocular infections. The paper by Romanowski et al. [1] reported that the most common species of coagulase-negative *staphylococci* to cause ocular infections was *Staphylococcus epidermidis*, with this species being isolated from ≥84% of endophthalmitis, ≥80% of keratitis and ≥62% of conjunctivitis/blepharitis caused by the coagulase-negative *staphylococci* group. The antibiotic profiles of these coagulase-negative staphylococci suggested that empirical treatments with vancomycin for endophthalmitis and cefazolin or vancomycin for keratitis were appropriate. Afzal et al. [2] found that of 63 *S. aureus* isolates from keratitis in USA or Australia, 87% of all the isolates were multidrug-resistant, and 17% of the isolates from microbial keratitis were extensively drug-resistant. Most Australian strains isolated from keratitis were susceptible to ciprofloxacin, but only 11% of the USA strains were. A follow-up study examining the virulence traits of these strains was published [3]. That study found no significant differences in the frequency of virulence genes between the strains isolated from infections (keratitis or conjunctivitis) compared to those isolated from non-infectious corneal infiltrative events. However, there were differences in the toxin genes produced by the strains isolated from keratitis and conjunctivitis. For example, conjunctivitis strains were more likely to possess genes encoding Panton–Valentine leukocidin. The fourth paper by Chen et al. [4] reported that hospital-acquired methicillin-susceptible *S. aureus* (MSSA) caused a significantly lower rate of keratitis but a higher rate of conjunctivitis than community-acquired MSSA. However, both types of MSSA were highly susceptible to several antibiotics, such as vancomycin and fluoroquinolones. It is worth noting that most centres reserve vancomycin to treat sight-threatening infections due to resistant organisms.

Orthokeratology is used in children to correct myopic refractive error during the day and control the development of myopia (short-sightedness) by modifying the shape of the cornea. These lenses are worn during sleep, which may increase the risk of ocular infection. A study by Chen et al. [5] found that guidelines for the best use of antibiotics during contact lens wear significantly improved prescribing habits, which, in turn, affected the compliance of patients with orthokeratology. Bacteria can respond to antibiotics in several ways, including activating their stress response processes. The study by Harshaw et al. [6] found that the antibiotics polymyxin B, cefazolin, ceftazidime and vancomycin that target the cell wall or cell membrane of *Serratia marcescens* activate the bacteria’s stress response, but antibiotics such as ciprofloxacin that do not act of the cell wall or membrane do not. The stress response may make bacteria tolerant to antibiotics, which may affect the outcomes of infection. A paper by Brothers et al. [7] also studied *S. marcescens*, examining the role of one of its transcription factors, EepR, in keratitis. The study found that mutants lacking *eepR* did not activate a cytokine response in corneal epithelial cells to the same degree as the wild-type strain, implicating EepR in producing pro-inflammatory mediators from *S. marcescens*.

Two papers examined the potential of new therapies for ocular infections. The paper by Romanowski et al. [8] reported on the use of predatory bacteria, *Bdellovibrio bacteriovorus* and *Micavibrio aeruginosavorus* to remove infecting *S. marcescens* or *Pseudomonas aeruginosa* from the eye. These predatory bacteria did not damage the eyes but were also unable to completely remove the infecting bacteria. The authors concluded that the predatory bacteria were no more effective than the normal host defense system at removing infecting bacteria from the eyes. The paper by Yasir et al. [9] examined the ability of antimicrobial peptides to reduce the ability of *S. aureus* to produce biofilms or to reduce the number of preformed biofilms. When used in conjunction with ciprofloxacin, the antimicrobial peptides resulted in substantial reductions in the number of bacteria in preformed biofilms or the ability of *S. aureus* to make biofilms. Importantly, *S. aureus* could not develop resistance to the antimicrobial peptides.

Finally, two papers examined new ways of preventing the microbial colonisation of contact lenses or contact lens cases. Reducing colonisation of lenses or cases might help reduce the incidence of contact lens-related keratitis. Dumpati et al. [10] demonstrated that short (thirty-second) exposure to ultraviolet light of 265 nm (UVC) could significantly reduce the number of *S. aureus* and *P. aeruginosa,* as well as the fungi *Candida albicans* and *Fusarium solani* adherent to contact lenses by up to 3.0 log_10_. Kalaiselvan et al. [11] reported that an antimicrobial contact lens produced by chemically binding the antimicrobial peptide Mel4 to etafilcon A contact lenses remained biocompatible. There were no significant differences in the clinical responses of an eye wearing the coated or non-coated lenses, nor were there differences in the comfort of the lenses during wear.

In conclusion, this Special Edition highlights important research on ocular infections, which may have consequences for prescribing antibiotics to treat ocular disease, as well as pathogenic mechanisms used by ocular pathogenic bacteria and new potential ways of preventing and treating ocular infections.
